# Increased salt intake does not worsen the progression of renal cystic disease in high water-loaded PCK rats

**DOI:** 10.1371/journal.pone.0207461

**Published:** 2019-03-14

**Authors:** Shizuko Nagao, Masanori Kugita, Kanako Kumamoto, Aya Yoshimura, Kazuhiro Nishii, Tamio Yamaguchi

**Affiliations:** 1 Education and Research Facility of Animal Models for Human Diseases, Fujita Health University, Toyoake, Aichi, Japan; 2 Faculty of Rehabilitation, School of Health Sciences, Fujita Health University, Toyoake, Aichi, Japan; 3 Department of Clinical Nutrition, Faculty of Health Science, Suzuka University of Medical Science, Suzuka, Mie, Japan; Istituto Di Ricerche Farmacologiche Mario Negri, ITALY

## Abstract

The anti-diuretic hormone arginine vasopressin is thought to be a detrimental factor in polycystic kidney disease (PKD). We previously reported that high water intake (HWI) reduced urine osmolality and urinary arginine vasopressin, improved renal function, and reduced the kidney/body weight ratio in PCK rats, an orthologous model of human PKD. In PKD patients, however, it is reported that HWI increases total kidney volume, urine volume, and urine sodium excretion, which could be a consequence of high salt intake. In the current study, we loaded PCK rats with high salt concurrently with HWI to determine whether this human-imitated condition exacerbates disease progression. PCK rats were assigned into 4 groups: control group (CONT: distilled water), HWI group (HWI: 5% glucose in water), HWI with 0.2% NaCl group (HWI+0.2%NaCl), and HWI with 0.45% NaCl group (HWI+0.45%NaCl). Total water intake during the experimental period was increased by 1.86-, 2.02-, and 2.42-fold in HWI, HWI+0.2%NaCl, and HWI+0.45%NaCl, and sodium intake was increased by 2.55- and 5.83-fold in HWI+0.2%NaCl and HWI+0.45%NaCl, respectively, compared with CONT. Systolic blood pressure was higher in HWI+0.2%NaCl and HWI+0.45%NaCl than in both CONT and HWI. Serum urea nitrogen, kidney/body weight ratio, cAMP, cystic area, and fibrosis index were significantly lower in HWI compared with CONT, and these ameliorative effects were not abrogated in either HWI+0.2%NaCl or HWI+0.45%NaCl. The amount of sodium excreted into the urine was increased by 2.50- and 8.38-fold in HWI+0.2%NaCl and HWI+0.45%NaCl, respectively, compared with HWI. Serum sodium levels were not different between the groups. These findings indicate that the beneficial effect of HWI against the progression of cystic kidney disease was not affected even by high salt-overload in this rodent model of PKD.

## Introduction

Polycystic kidney disease (PKD) is the most common hereditary renal disorder with countless cysts in bilateral kidneys. The stimulation of cell proliferation and fluid secretion in the renal tubule epithelium are the major components of cyst development [1, 2]. Mutations of the responsible genes for autosomal dominant PKD and autosomal recessive PKD are thought to cause intracellular calcium reduction [3–5], and a cAMP-mediated cell proliferation phenotype is induced by this reduction of calcium [6]. Arginine vasopressin (AVP) is speculated to be a detrimental factor for the progression of PKD by stimulating cell proliferation through the AVP V_2_ receptor with the downstream accumulation of intracellular cAMP, which stimulates the B-Raf/MEK/ERK signaling pathway in animal models and humans [7–9]. Several therapeutic strategies to inhibit the effect of AVP have been developed recently. One pharmacological approach is to block the V_2_ receptor to inhibit the effect of secreted AVP [10, 11], while a simpler approach is to use excessive water drinking to reduce the release of AVP into the blood [12, 13]. We previously reported that high water intake (HWI) via enhanced hydration by adding 5% glucose to drinking water had an inhibitory effect on the progression of PKD with reduced urine osmolality and urinary AVP in PCK rats, a human gene orthologous model of autosomal recessive PKD [14]. In addition, HWI by feeding with a hydrated agar diet also ameliorated disease progression in PCK rats [15]. Whereas in patients with autosomal dominant PKD, HWI did not ameliorate total kidney volume and kidney function slopes, and rather, those parameters became worse in a 1-year non-randomized study, although their blood AVP level was decreased [16]. In that case, increased urine sodium excretion was observed, which could possibly be the consequence of primary high salt intake, probably because if the patients did not increase their sodium intake, the plasma sodium level may be reduced inappropriately by HWI. This clinical outcome suggests that increased salt intake may cause the deterioration of symptoms in PKD patients. Therefore, in this study, in order to determine whether increased salt intake attenuates the ameliorative effect of HWI, we loaded PCK rats with high sodium chloride (NaCl) concurrently with HWI.

## Materials and methods

### Animals and study design

The PCK rat strain was originally derived from a Sprague-Dawley colony in Charles River Japan (Tokyo, Japan). A splicing mutation with subsequent skipping of exon 36 and a frameshift in the human orthologous Pkhd1 gene causes renal cysts originating from the collecting ducts and congenital hepatic fibrosis complicated with biliary cysts [14, 17–19]. Sprague-Dawley rats (males), also obtained from Charles River Japan, were used as normal control (wild-type) animals.

The rats were bred and maintained at the Education and Research Facility of Animal Models for Human Diseases, Fujita Health University. All animals used in the current study were allowed free access to laboratory chow and water with or without glucose and/or NaCl. We randomly assigned 3.5-week-old male PCK rats (n = 20) and Sprague-Dawley rats (n = 12) to 4 groups: control group (CONT: distilled water), HWI group (HWI: 5% glucose), HWI with 0.2% NaCl-loaded group (HWI+0.2%NaCl), or HWI with 0.45% NaCl-loaded group (HWI+0.45%NaCl), which were treated from 4 to 20 weeks of age. Food consumption was measured every week.

At the age of 19 weeks, systolic blood pressure (SBP) was determined using a tail-cuff sphygmomanometer (BP98A; Softron Co., Ltd., Tokyo, Japan) [18, 19]. At 19.5 weeks of age, 24-h urine samples were collected in metabolic cages and urine volume was measured. At 20 weeks of age, after body weight measurement, the animals were anesthetized with isoflurane (Pfizer, Inc., New York, NY, USA), and both kidneys were removed rapidly, causing lethal exsanguination.

### Ethics statement

The rats were handled ethically according to the Regulations for the Management of Laboratory Animals at Fujita Health University. The experimental protocol for the ethical use of these animals was approved by the Animal Care and Use Committee at Fujita Health University (Permit No.: AP16079).

### Analysis of urine and blood for electrolytes and proteins

Serum samples were collected for measurements of serum urea nitrogen (SUN), creatinine, sodium, chloride, potassium, inorganic phosphorus, and calcium. Urine samples were measured for osmolality, sodium, chloride, potassium, inorganic phosphorus, and calcium. Serum and urine biochemical parameters were analyzed by Oriental Yeast Co., Ltd. (Tokyo, Japan). Urine and biochemical parameters were measured according to the manufacturers’ instructions and are expressed as the mean ± standard deviation (SD).

### Serum aldosterone

Serum aldosterone measurement was performed with an enzyme immunoassay (#ADI-900-173; Enzo Life Sciences, Inc., Farmingdale, NY, USA).

### Analysis of renal tissue cAMP content

To measure renal tissue cAMP content, kidneys were prepared by homogenization with HCl. Extracted cAMP levels were analyzed by using an enzyme-linked immunosorbent assay kit (#ADI-900-163; Enzo Life Sciences, Inc., New York, NY, USA), and were corrected by protein concentration. Outcome values are expressed as mean ± SD values.

### Histopathological analyses

Total wet kidney weight was measured, and the right kidney was sectioned and immersed in 4% paraformaldehyde for histopathological analyses. Kidney sections were stained with hematoxylin and eosin in each group. Cystic area was measured in five random fields (×100 magnification) of hematoxylin and eosin (H&E)-stained sections, and fibrosis index was measured from five random fields (×100 magnification) of picrosirius red-stained kidney sections. Cystic area and fibrosis index (% of total field) were measured by a blinded observer using WinROOF software version 6.4 (Mitani Corporation, Fukui, Japan) and are expressed as the mean ± SD.

### Statistical analysis

The normality of sample distributions was tested and confirmed by the Shapiro-Wilk normality test. Pairwise comparisons between the CONT group and the other treatment groups were made using Welch’s t-test. The multiplicity of hypothesis testing was adjusted by Holm’s method [20]. Power analysis was performed for the two-sample t-test to select the sample size that could detect a pre-specified effect size or observed difference in means with a significance level of 0.05 and power of 0.8. The monotone trend in terms of group order was tested by the Jonckheere-Terpstra trend test. All P-values were two-tailed, and P < 0.05 was considered to be statistically significant.

### Correlation analysis

We analyzed the correlations between two factors selected from water intake, urine volume, urine osmolality, sodium intake, urine sodium excretion (U-Na), urine chlorine excretion (U-Cl), kidney per body weight ratio (KB%), SBP, or SUN either in PCK or Sprague-Dawley rats by the Pearson correlation coefficient, and a correlation was considered significant at P < 0.05.

## Results

### Effect of HWI and NaCl overload on body weight and total intake of food, water, and sodium

#### Body weight at the end of the experiment

**Fig 1:**
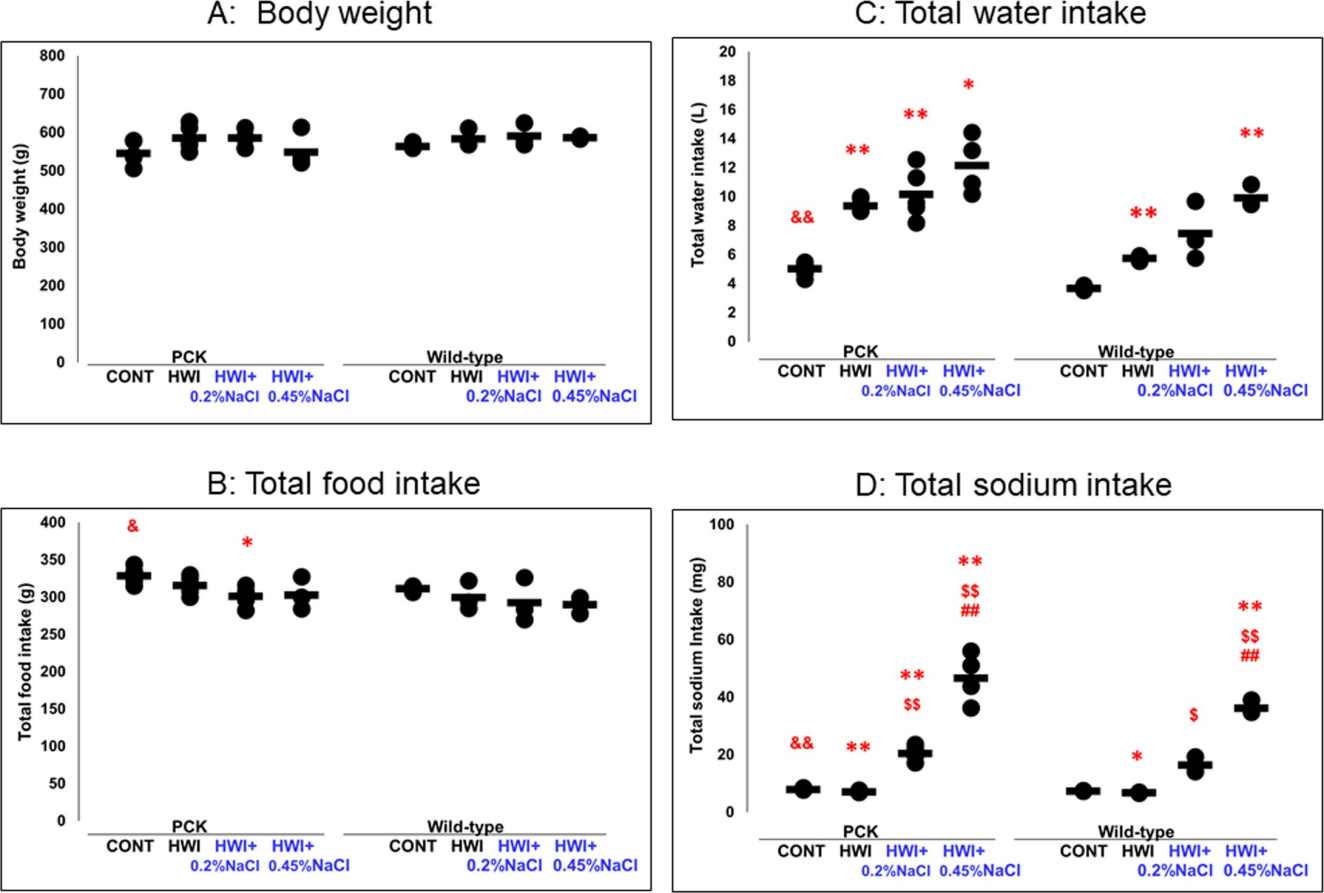
Effects of HWI and NaCl overload on body weight and total intake of food, water, and sodium. CONT, distilled water group; HWI, high water intake group (5% glucose); HWI+0.2%NaCl, high water intake with 0.2% NaCl group; HWI+0.45%NaCl, high water intake with 0.45% NaCl group. Difference between PCK CONT and wild-type CONT groups: &, *P* < 0.05; &&, *P* < 0.01. Comparison between CONT and treated groups: *1, *P* = 0.050; *, *P* < 0.05; **, *P* < 0.01. Comparison between HWI and HWI+0.2%NaCl or HWI+0.45%NaCl: $, *P* < 0.05; $$, *P* < 0.01. Comparison between HWI+0.2%NaCl and HWI+0.45%NaCl: ##, *P* < 0.01.

In PCK rats with polycystic kidneys, final body weight was not different in all groups (Fig 1A). In wild-type rats with normal kidneys, body weight was not different in all groups. In the animals without HWI (CONT group), body weight was not different between PCK rats and wild-type rats.

#### Total food intake during the experimental period

In PCK rats, total food intake was decreased by 8% only in HWI+0.2%NaCl (301 ± 13 g, *P* < 0.05) compared with CONT (329 ± 10 g) (Fig 1B). No difference was shown between the other groups. In wild-type rats, there was no difference between the groups. In the animals without HWI (CONT group), total food intake was increased in PCK rats (*P* < 0.05) compared with wild-type rats (311 ± 5 g).

#### Total water intake during the experimental period

In PCK rats, total water intake was increased by 1.87-, 2.02-, and 2.42-fold in HWI (9.38 ± 0.50 L, *P* < 0.01), HWI+0.2%NaCl (10.18 ± 1.74 L, *P* < 0.01), and HWI+0.45%NaCl (12.18 ± 1.97 L, *P* < 0.05), respectively, compared with CONT (5.03 ± 0.47 L) (Fig 1C). In wild-type rats, total water intake was increased by 1.56-, 2.02-, and 2.68-fold in HWI (5.77 ± 0.21 L, *P* < 0.01), HWI+0.2%NaCl (7.47 ± 2.01 L, not significant [NS]), and HWI+0.45%NaCl (9.94 ± 0.78 L, *P* < 0.01), respectively, compared with CONT (3.70 ± 0.18 L). No difference was shown between HWI and salt-loaded HWI rats. In the animals without HWI (CONT group), total water intake was increased in PCK rats (*P* < 0.01) compared with wild-type rats.

#### Total sodium intake during the experimental period

In PCK rats, total sodium intake was decreased by 0.89-fold in HWI (7.2 ± 0.3 mg, *P* < 0.01) compared with CONT (8.0 ± 0.3 mg) (Fig 1D). By NaCl loading, total sodium intake was increased by 2.55- and 5.83-fold in HWI+0.2%NaCl (20.4 ± 2.5 mg, *P* < 0.01) and HWI+0.45%NaCl (46.7 ± 8.6 mg, *P* < 0.01), respectively, compared with CONT. In addition, total sodium intake was increased by 2.85- and 6.51-fold in HWI+0.2%NaCl (*P* < 0.01) and HWI+0.45%NaCl (*P* < 0.01), respectively, compared with HWI. Between the salt-loaded HWI groups, the total amount of sodium intake was higher in HWI+0.45%NaCl (*P* < 0.01) than in HWI+0.2%NaCl. In wild-type rats, a similar trend was shown, in which total sodium intake was decreased by 0.93-fold in HWI (6.8 ± 0.2 mg, *P* < 0.05) compared with CONT (7.3 ± 0.1 mg). Total sodium intake was increased by 2.25- and 4.94-fold in HWI+0.2%NaCl (16.5 ± 2.6 mg, *P* = 0.05) and HWI+0.45%NaCl (36.3 ± 2.3 mg, *P* < 0.01), respectively, compared with CONT. Total sodium intake was increased by 2.42- and 5.31-fold in HWI+0.2%NaCl (*P* < 0.05) and HWI+0.45%NaCl (*P* < 0.01), respectively, compared with HWI. In HWI+0.45%NaCl (*P* < 0.01), total sodium intake was increased compared with HWI+0.2%NaCl. In the animals without HWI (CONT group), it was increased in PCK rats (*P* < 0.01) compared with wild-type rats.

### Effect of HWI and NaCl overload on urine volume and osmolality

#### Urine volume

**Fig 2:**
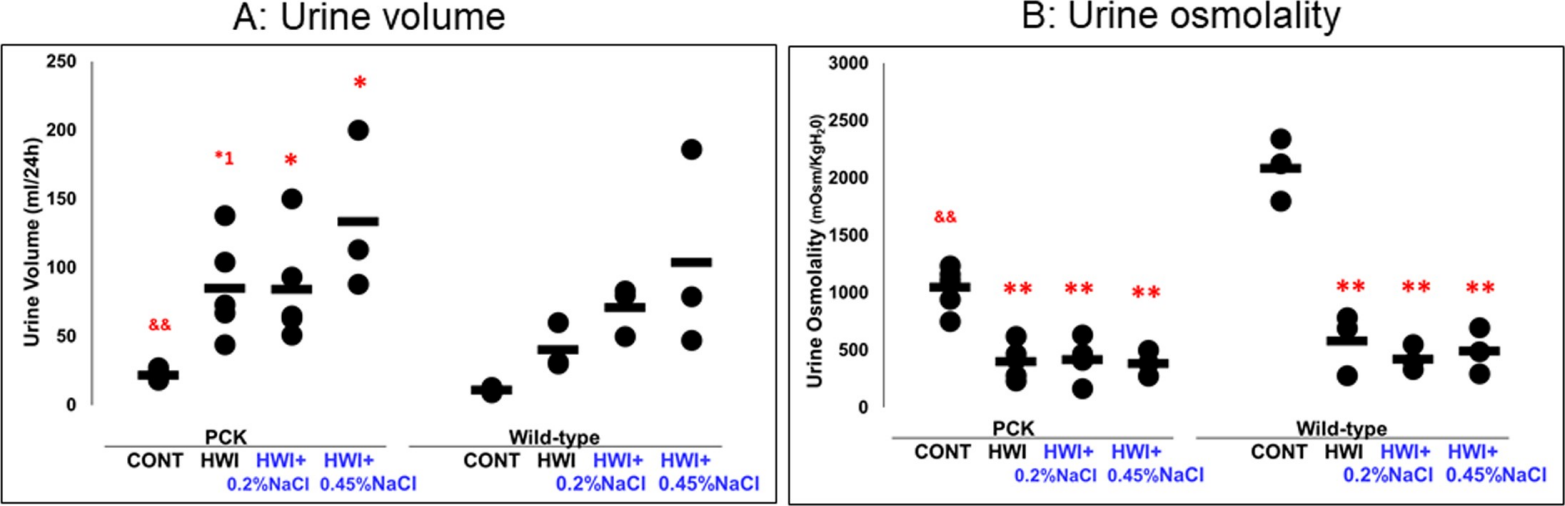
Effects of HWI and NaCl overload on urine volume and osmolality. CONT, distilled water group; HWI, high water intake group (5% glucose); HWI+0.2%NaCl, high water intake with 0.2% NaCl group; HWI+0.45%NaCl, high water intake with 0.45% NaCl group. Difference between PCK CONT and wild-type CONT groups: &&, *P* < 0.01. Comparison between CONT and treated groups: *1, *P* = 0.052; *, *P* < 0.05; **, *P* < 0.01.

In PCK rats, urine volume was increased by 3.90-, 3.87-, and 6.12-fold in HWI (85 ± 36 mL/24 h, *P* = 0.052), HWI+0.2%NaCl (84 ± 40 mL/24 h, *P* < 0.05), and HWI+0.45%NaCl (134 ± 59 mL/24 h, P < 0.05), respectively, compared with CONT (22 ± 3 mL/24 h) (Fig 2A). In wild-type rats, urine volume was not different between the groups. In the animals without HWI (CONT group), it was increased in PCK rats (*P* < 0.01) compared with wild-type rats (11 ± 2 mL/24 h).

#### Urine osmolality

In PCK rats, urine osmolality was decreased by 0.38-, 0.40-, and 0.37-fold in HWI (402 ± 155 mOsm/KgH_2_O, *P* < 0.01), HWI+0.2%NaCl (421 ± 168 mOsm/KgH_2_O, *P* < 0.01), and HWI+0.45%NaCl (387 ± 112 mOsm/KgH_2_O, *P* < 0.01), respectively, compared with CONT (1050 ± 176 mOsm/KgH_2_O) (Fig 2B). In wild-type rats, urine osmolality was decreased by 0.28-, 0.20-, and 0.24-fold in HWI (584 ± 270 mOsm/KgH_2_O, *P* < 0.01), HWI+0.2%NaCl (424 ± 112 mOsm/KgH_2_O, *P* < 0.01), and HWI+0.45%NaCl (493 ± 200 mOsm/KgH_2_O, *P* < 0.01), respectively, compared with CONT (2088 ± 274 mOsm/KgH_2_O). Urine volume and osmolality were unaffected by overload with high salt in both PCK and wild-type rats with HWI. In the animals without HWI (CONT group), it was decreased in PCK rats (*P* < 0.01) compared with wild-type rats.

### Effect of HWI and NaCl overload on SBP, aldosterone, SUN, creatinine, KB%, cAMP, and renal histology

#### SBP

**Fig 3:**
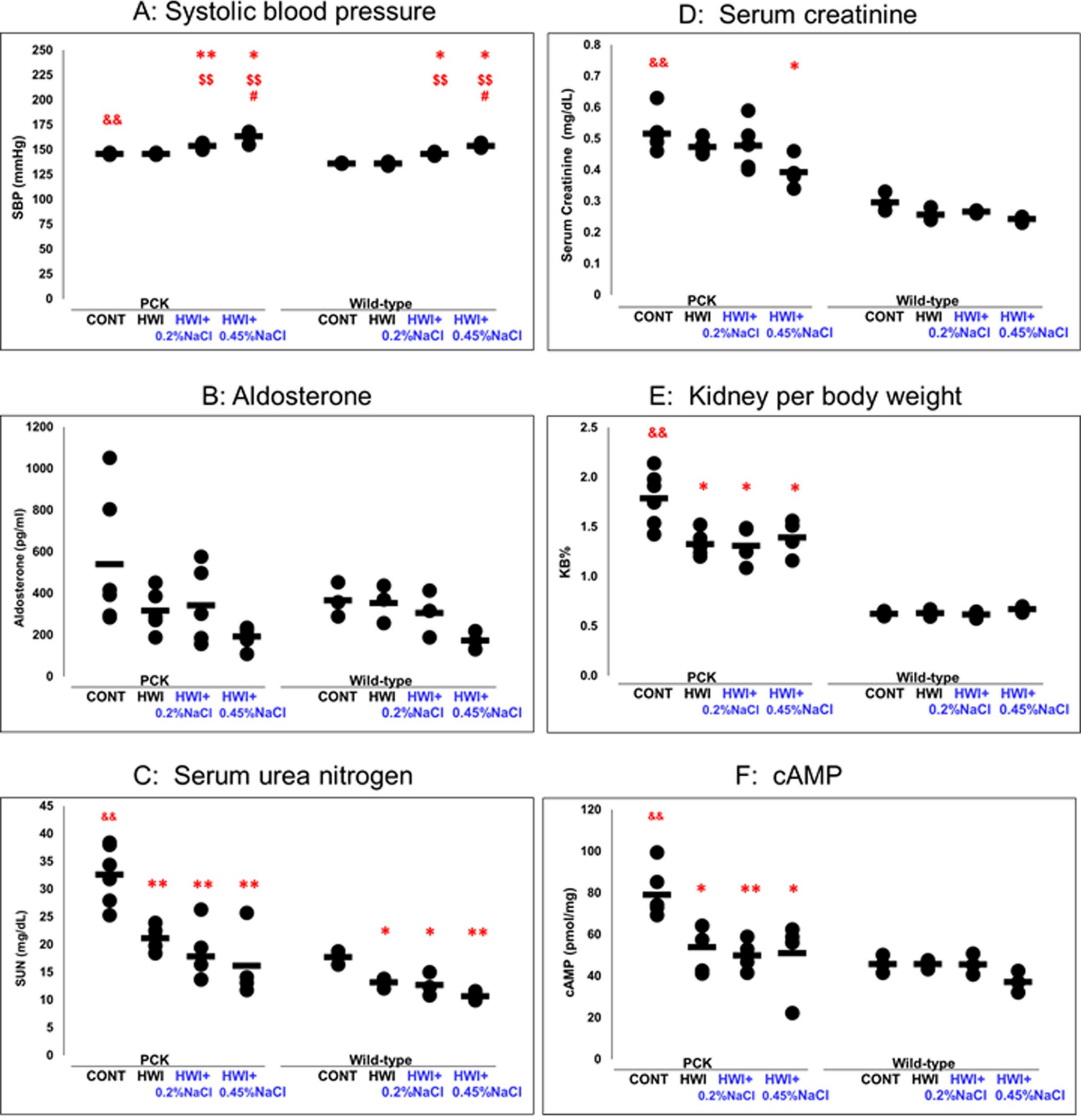
Effects of HWI and NaCl overload on SBP, aldosterone, SUN, serum creatinine, KB%, and renal cAMP. CONT, distilled water group; HWI, high water intake group (5% glucose); HWI+0.2%NaCl, high water intake with 0.2% NaCl group; HWI+0.45%NaCl, high water intake with 0.45% NaCl group. Difference between PCK CONT and wild-type CONT groups: &&, *P* < 0.01. Comparison between CONT and treated groups: *, *P* < 0.05; **, *P* < 0.01. Comparison between HWI and HWI+0.2%NaCl or HWI+0.45%NaCl: $$, *P* < 0.01. Comparison between HWI+0.2%NaCl and HWI+0.45%NaCl: #, *P* < 0.05.

In PCK rats, SBP was higher in HWI+0.2%NaCl (154 ± 3 mmHg, *P* < 0.01) and HWI+0.45%NaCl (164 ± 6 mmHg, *P* < 0.05) compared with CONT (146 ± 1 mmHg) (Fig 3A). SBP was higher in HWI+0.2%NaCl (*P* < 0.01) and HWI+0.45%NaCl (*P* < 0.01) compared with HWI (146 ± 1 mmHg), and it was higher in HWI+0.45%NaCl (*P* < 0.05) compared with HWI+0.2%NaCl. In wild-type rats, SBP was higher in HWI+0.2%NaCl (146 ± 2 mmHg, *P* < 0.05) and HWI+0.45%NaCl (154 ± 3 mmHg, *P* < 0.05) compared with CONT (136 ± 1 mmHg). SBP was higher in HWI+0.2%NaCl (*P* < 0.01) and HWI+0.45%NaCl (*P* < 0.01) compared with HWI (136 ± 2 mmHg), and it was higher in HWI+0.45%NaCl (*P* < 0.05) compared with HWI+0.2%NaCl. In the animals without HWI (CONT group), it was increased in PCK rats (*P* < 0.01) compared with wild-type rats.

#### Aldosterone

There was no difference in aldosterone between the groups for PCK and wild-type rats (Fig 3B).

#### SUN

In PCK rats, SUN was significantly lower in HWI (21.2 ± 2.2 mg/dL, *P* < 0.01), HWI+0.2%NaCl (17.9 ± 5.3 mg/dL, *P* < 0.01), and HWI+0.45%NaCl (16.2 ± 6.4 mg/dL, *P* < 0.01) compared with CONT (32.6 ± 5.3 mg/dL) (Fig 3C). In wild-type rats, SUN was significantly lower in HWI (13.2 ± 1.0 mg/dL, *P* < 0.05), HWI+0.2%NaCl (12.7 ± 2.1 mg/dL, *P* < 0.05), and HWI+0.45%NaCl (10.7 ± 0.9 mg/dL, *P* < 0.01) compared with CONT (17.7 ± 1.2 mg/dL). In the animals without HWI (CONT group), it was increased in PCK rats (*P* < 0.01) compared with wild-type rats.

#### Serum creatinine

In PCK rats, serum creatinine was significantly lower in HWI+0.45%NaCl (0.39 ± 0.05 mg/dL, *P* < 0.05) compared with CONT (0.52 ± 0.06 mg/dL) (Fig 3D). In wild-type rats, there was no difference between the groups. In the animals without HWI (CONT group), it was increased in PCK rats (*P* < 0.01) compared with wild-type rats (0.30 ± 0.03 mg/dL).

#### KB%

In PCK rats, KB% was significantly lower in HWI (1.33 ± 0.13%, *P* < 0.05), HWI+0.2%NaCl (1.31 ± 0.17%, *P* < 0.05), and HWI+0.45%NaCl (1.40 ± 0.18%, *P* < 0.05) compared with CONT (1.79 ± 0.27%) (Fig 3E). In wild-type rats, KB% was not different between the groups. In the animals without HWI (CONT group), KB% was increased in PCK rats (P < 0.01) compared with wild-type rats (0.62 ± 0.03%).

#### Renal cAMP

In PCK rats, renal cAMP was significantly lower in HWI (53.9 ± 11.3 pmol/mg, *P* < 0.05), HWI+0.2%NaCl (50.0 ± 6.5 pmol/mg, *P* < 0.01), and HWI+0.45%NaCl (51.2 ± 16.4 pmol/mg, *P* < 0.05) compared with CONT (79.1 ± 11.3 pmol/mg) (Fig 3F). In wild-type rats, renal cAMP was not different between the groups. In the animals without HWI (CONT group), it was increased in PCK rats (*P* < 0.01) compared with wild-type rats (45.8 ± 4.4 pmol/mg).

#### Renal histology

**Fig 4:**
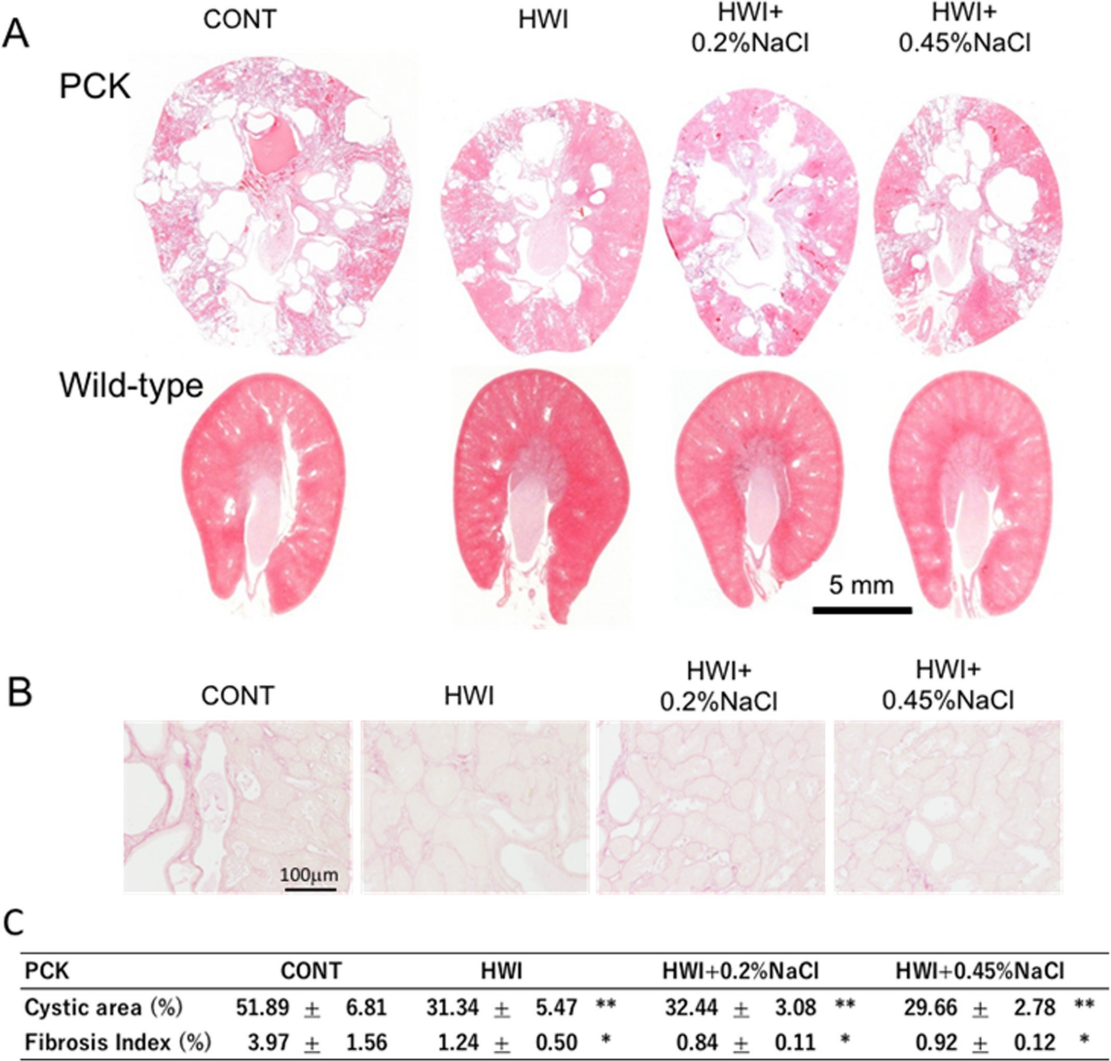
Effect of HWI and NaCl overload on renal histology, cystic area, and fibrosis index. (A) H&E-stained kidney sections of PCK and wild-type rats. (B) Picrosirius red-stained kidney sections of PCK rats. (C) Cystic area and fibrosis index obtained from H&E-stained and picrosirius red-stained kidney sections, respectively, in each group. CONT, distilled water group; HWI, high water intake group (5% glucose); HWI+0.2%NaCl, high water intake with 0.2% NaCl group; HWI+0.45%NaCl, high water intake with 0.45% NaCl group. Comparison between CONT and treated groups: *, *P* < 0.05; **, *P* < 0.01.

In H&E-stained kidney sections of PCK rats, the number of cysts was decreased, while the portion of normal tissue was increased in HWI, HWI+0.2%NaCl, and HWI+0.45%NaCl compared with CONT. In wild-type rats, no difference was shown between the groups (Fig 4A).

#### Cystic area and fibrosis index

In PCK rats, the portion of cystic area per cross section was significantly decreased in HWI (31.3 ± 5.5, *P* < 0.01), HWI+0.2%NaCl (32.4 ± 3.1, *P* < 0.01), and HWI+0.45%NaCl (29.7 ± 2.8, *P* < 0.01) compared with CONT (51.9 ± 5.1) (Fig 4A and 4C), and the fibrosis index was significantly lower in HWI (1.24 ± 0.50, *P* < 0.05), HWI+0.2%NaCl (0.84 ± 0.11, *P* < 0.05), and HWI+0.45%NaCl (0.92 ± 0.12, *P* < 0.05) compared with CONT (3.97 ± 1.56) (Fig 4B and 4C). Therefore, the beneficial effects of HWI on renal tissue were not diminished by high salt overload in PCK rats as measured histologically.

### Effect of HWI and NaCl overload on urine excretion and blood concentrations of electrolytes

#### Total U-Na during the experimental period and serum sodium concentration (S-Na)

**Fig 5:**
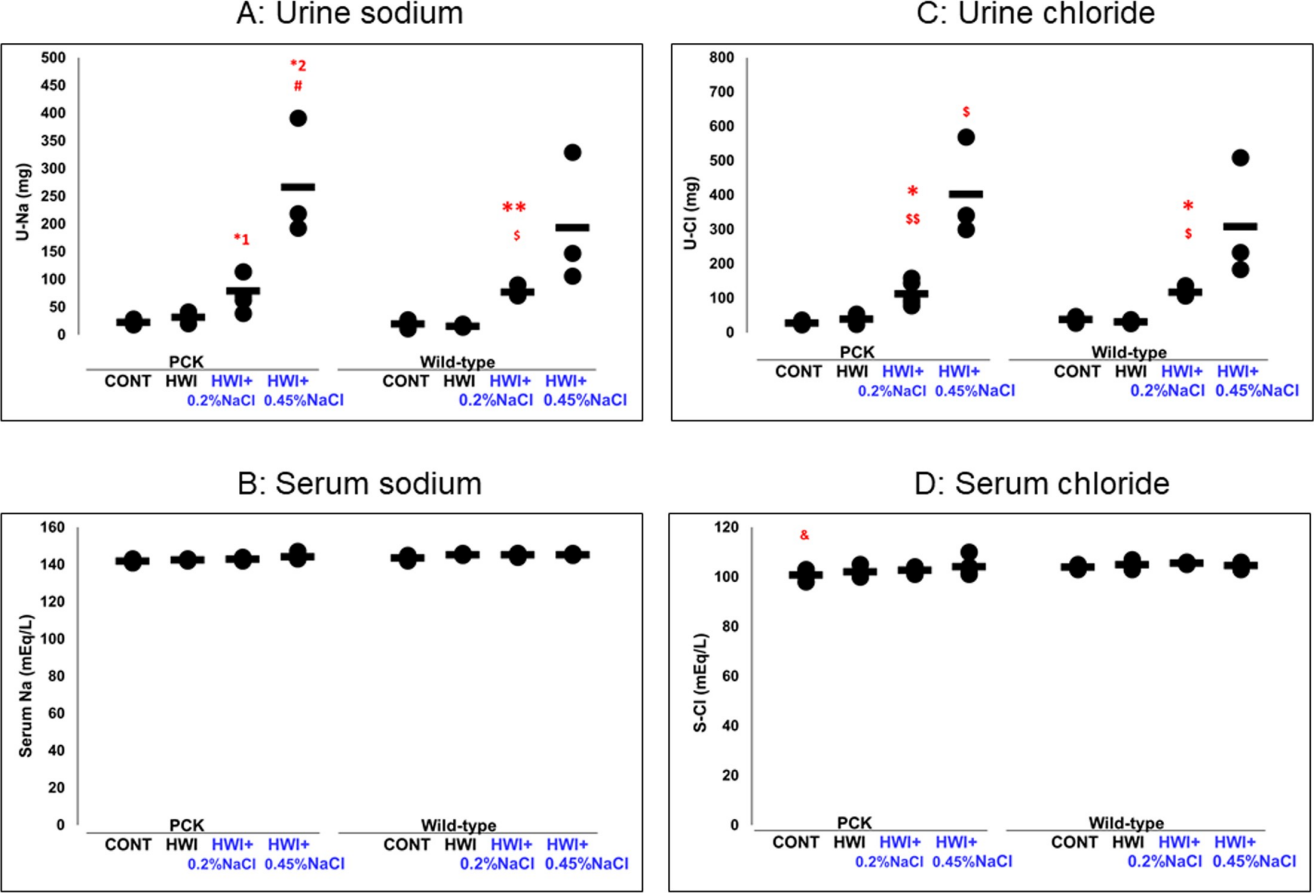
Effects of HWI and salt intake on urine excretion and serum concentrations of NaCl. CONT, distilled water group; HWI, high water intake group (5% glucose); HWI+0.2%NaCl, high water intake with 0.2% NaCl group; HWI+0.45%NaCl, high water intake with 0.45% NaCl group. Difference between PCK CONT and wild-type CONT groups: &, *P* < 0.05. Comparison between CONT and treated groups: *1, *P* = 0.053; *2, *P* = 0.059; *, *P* < 0.05; **, P < 0.01. Comparison between HWI and HWI+0.2%NaCl or HWI+0.45%NaCl: $, *P* < 0.05; $$, *P* < 0.01. Comparison between HWI+0.2%NaCl and HWI+0.45%NaCl: #, *P* < 0.05.

The total amount of U-Na was increased by 3.52- and 11.82-fold in HWI+0.2%NaCl (80 ± 33 mg, *P* = 0.053) and HWI+0.45%NaCl (267 ± 108 mg, *P* = 0.059), respectively, compared with CONT (23 ± 4 mg), and it was increased by 2.50-fold in HWI+0.2%NaCl (NS) and 8.38-fold in HWI+0.45%NaCl (NS) compared with HWI (32 ± 8 mg), (Fig 5A). In wild-type rats, U-Na was increased by 3.90- and 9.75-fold in HWI+0.2%NaCl (78 ± 11 mg, *P* < 0.01) and HWI+0.45%NaCl (194 ± 119 mg, NS), respectively, compared with CONT (20 ± 8 mg), and it was increased by 4.83- and 12.08-fold in HWI+0.2%NaCl (*P* < 0.05) and HWI+0.45%NaCl (NS), respectively, compared with HWI (16 ± 3 mg).

S-Na was not different between the groups in either PCK or wild-type rats (Fig 5B).

#### Total U-Cl during the experimental period and serum chloride concentration (S-Cl)

In PCK rats, the total amount of U-Cl was increased by 1.44-, 4.03-, and 14.38-fold in HWI (40 ± 11 mg, NS), HWI+0.2%NaCl (113 ± 36 mg, *P* < 0.05), and HWI+0.45%NaCl (403 ± 144 mg, NS), respectively, compared with CONT (28 ± 5 mg). U-Cl was increased in HWI+0.2%NaCl (*P* < 0.01) and HWI+0.45%NaCl (*P* < 0.05) compared with HWI (Fig 5C). In wild-type rats, U-Cl was increased by 3.08-fold in HWI+0.2%NaCl (117 ± 16 mg, *P* < 0.05) compared with CONT (38 ± 10 mg). U-Cl was increased in HWI+0.2%NaCl (*P* < 0.05) compared with HWI (31 ± 6 mg). S-Cl was not different between all groups in PCK and wild-type rats (Fig 5D). In the animals without HWI (CONT group), it was decreased in PCK rats (100 ± 2 mEq/L, *P* < 0.05) compared with wild-type rats (104 ± 1 mEq/L).

#### Total urine potassium excretion (U-K) during the experimental period and serum potassium concentration (S-K)

The total amount of U-K was not different between all groups of PCK rats (Table 1). U-K was not different between all groups of wild-type rats, except between HWI+0.45%NaCl (123 ± 18 mg, *P* < 0.05) and HWI (83 ± 15 mg). In PCK rats, S-K was lower in HWI+0.2%NaCl (4.68 ± 0.19 mEq/L, *P* < 0.05) compared with CONT (5.33 ± 0.37 mEq/L) (Table 1). In wild-type rats, S-K was not different between all groups, except between HWI+0.45%NaCl (4.40 ± 0.10 mEq/L, *P* < 0.05) and CONT (5.07 ± 0.15 mEq/L).

**Table 1:** Effect of HWI and NaCl overload on urine excretion and blood concentrations of electrolytes.

PCK		CONT				HWI				HWI+0.2%NaCl					HWI+0.45%NaCl			
U-K (mg)	135	±	19		142	±	20		127	±	19			118	±	37		
S-K (mEq/L)	5.33	±	0.37		5.28	±	0.44		4.68	±	0.19	**	$	4.85	±	0.37		
U-IP (mg)	23.3	±	5.7	&&	29.3	±	5.2		25.6	±	6.8			31.3	±	2.8	*	
S-IP (mEq/L)	7.33	±	0.24		6.66	±	0.48	*	6.48	±	0.22	**		6.20	±	0.64	*	
U-Ca (mg)	2.13	±	0.61	&&	2.54	±	1.25		3.89	±	1.39	*		4.84	±	2.73		
S-Ca (mEq/L)	10.7	±	0.4	&	10.5	±	0.3		10.5	±	0.3			10.3	±	0.3		
Wild-type		CONT								HWI+0.2%NaCl					HWI+0.45%NaCl			
U-K (mg)	108	±	19		83	±	15		118	±	8		$	123	±	18		$
S-K (mEq/L)	5.07	±	0.15		4.93	±	0.21		5.03	±	0.67			4.40	±	0.10	**	$
U-IP (mg)	3.2	±	1.6		9.7	±	1.8	*	18.2	±	2.9	**	$	17.2	±	1.1	**	$$
S-IP (mEq/L	6.60	±	0.70		6.87	±	0.45		6.60	±	0.62			6.33	±	0.40		
U-Ca (mg)	0.50	±	0.26		0.47	±	0.09		1.16	±	0.40			1.96	±	0.84		
S-Ca (mEq/L)	9.8	±	0.3		10.0	±	0.2		10.0	±	0.2			10.0	±	0.1		

The parameters are expressed as the mean ± SD. Difference between PCK CONT and wild-type CONT groups:

&, *P* < 0.05;

&&, *P* < 0.01. Comparison between CONT and treated groups: *1, *P* = 0.052; *, *P* < 0.05; **, *P* < 0.01. Comparison between HWI and HWI+0.2%NaCl or HWI+0.45%NaCl:

$, *P* < 0.05.

#### Total urine inorganic phosphorus excretion (U-IP) during the experimental period and serum inorganic phosphorus concentration (S-IP)

In PCK rats, the total amount of U-IP was higher only in HWI+0.45%NaCl (31.3 ± 2.8 mg, *P* = 0.052) compared with CONT (23.3 ± 5.7 mg) (Table 1). In wild-type rats, it was significantly increased in HWI (9.7 ± 1.8 mg, *P* < 0.05), HWI+0.2%NaCl (18.2 ± 2.9 mg, *P* < 0.01), and HWI+0.45%NaCl (17.2 ± 1.1 mg, *P* < 0.01) compared with CONT (3.2 ± 1.6 mg). In the animals without HWI (CONT group), U-IP was increased in PCK rats (*P* < 0.01) compared with wild-type rats.

In PCK rats, S-IP was lower in HWI+0.2%NaCl (6.48 ± 0.22 mEq/L, *P* < 0.01), and HWI+0.45%NaCl (6.20 ± 0.64 mEq/L, *P* < 0.05) compared with CONT (7.33 ± 0.24 mEq/L). In wild-type rats, there was no difference between the groups.

#### Total urine calcium excretion (U-Ca) during the experimental period and serum calcium concentration (S-Ca)

U-Ca was not different between all groups in both PCK and wild-type rats. S-Ca was not different between all groups in both PCK and wild-type rats (Table 1). In the animals without HWI (CONT group), U-Ca was increased in PCK rats (2.13 ± 0.61 mg, *P* < 0.01) compared with wild-type rats (0.50 ± 0.26 mg).

### Correlation between two factors in PCK and wild-type rats

Correlations were observed between water intake and urine volume, urine volume and osmolality, sodium intake and water intake, sodium intake and U-Na, U-Na and urine volume, and U-Na and U-Cl in PCK and wild-type rats (Table 2). While no correlation was observed between KB% and sodium intake or KB% and U-Na either in PCK or wild-type rats. Although SBP was correlated with sodium intake, SBP was not correlated with KB% in PCK or wild-type rats.

**Table 2:** Correlation between two factors in PCK and wild-type rats.

Correlation Factor		PCK		Wild-type	
		R	*P*-value	R	*P*-value
Water Intake	Urine Volume	0.768	0.000	0.769	0.003
Urine Volume	Osmolality	-0.828	0.000	-0.666	0.018
Sodium Intake	Water Intake	0.744	0.000	0.892	0.000
Sodium Intake	U-Na	0.926	0.000	0.869	0.000
U-Na	Urine Volume	0.762	0.000	0.920	0.000
U-Na	U-Cl	0.997	0.000	0.999	0.000
KB%	SUN	0.789	0.000	-0.492	NS
KB%	Sodium Intake	-0.271	NS	0.554	NS
KB%	U-Na	-0.337	NS	0.566	NS
SBP	Sodium Intake	0.948	0.000	0.960	0.000
SBP	KB%	-0.263	NS	0.507	NS

## Discussion and Conclusion

Our previous study showed that HWI reduced KB% and cystic area in kidney cross sections with a reduction of SUN, urinary AVP, urine osmolality, and the number of AVP V_2_ receptor-, B-Raf-, P-ERK-, and PCNA-positive renal cells in PCK rats [14]. Hopp et al. also reported that HWI treatment with 1% agar decreased cyst area, KB%, plasma urea, creatinine, P-ERK and PCNA expression, and urinary AVP [15], confirming that increased water intake slows down the progression of renal cystic disease in this rat model. Our current results have shown that the ameliorative effect of HWI on SUN, KB%, cystic area, and fibrosis index was not diminished by overload with high salt. Serum creatinine was decreased in HWI+0.45%NaCl compared with HWI, which also suggests that sodium loading does not worsen disease progression. It is evident that the secretion of sodium and chloride into the urine was increased by NaCl intake; however, serum sodium and chloride concentrations were not increased. This suggests that the ability of the kidney to excrete NaCl into the urine was sufficiently maintained in the current disease stage of PCK rats.

SBP was higher in PCK rats compared with wild-type rats in the current study, as well as in our previous study [18]. The consequence of hypertension on renal cystic disease in PCK rats has not been substantially elucidated, and whether the increased blood pressure may be caused spontaneously by a *Pkhd1* gene mutation or caused secondarily by a reduction of renal function in PCK rats is unknown. One possible mechanistic hypothesis for the increased blood pressure in this model may be enhanced sodium reabsorption through epithelial Na+ channels in the collecting duct, since Kaimori et al. recently showed that mutation of the *Pkhd1* gene in PCK rats alters the cellular localization of the ubiquitin ligase Nedd4-2, which increases the expression of apical epithelial Na+ channels to stimulate sodium reabsorption into the blood [21]. Our current results have shown that even NaCl overload in this rat model further increased blood pressure, but the renal protective effect of HWI was not affected. It is of note that the ratio of blood pressure increase by sodium overload in PCK rats was similarly observed in wild-type rats, and renal function and histology were not evidently affected in either PCK or wild-type rats, showing that the detrimental effect of sodium loading in rat kidneys was not obvious in the present study. Further, the plasma level of aldosterone, a component of the renin angiotensin aldosterone system (RAAS), was not affected by high salt loading in animals with or without HWI, supporting the hypothesis that increased salt intake may not be able to alter the RAAS significantly in the current study. Generally, aldosterone promotes the reabsorption of sodium in the kidney, increases the blood circulation volume, and raises blood pressure. Storing sodium in the body decreases aldosterone secretion. In contrast, in salt-sensitive hypertension, Shibata et al. reported that increased sodium intake activates the mineralocorticoid receptor by activating the small G protein RAS-related C3 botulinum toxin substrate 1 (Rac1), which activates body sodium storage to cause high blood pressure [22]. In the current study, in both PCK and wild-type rats, blood pressure and sodium excretion into urine were increased by salt loading, whereas aldosterone concentration was not changed significantly. Therefore, salt-sensitive hypertension may have been created in PCK and wild-type rats in the present study without a distinct alteration of the RAAS. There is a possible explanation why high salt loading plus HWI did not have an inferior effect compared to HWI alone. Despite increased sodium intake, the suppression of AVP release by HWI may lead to decreased levels of renal cAMP, which could be sufficient to ameliorate disease progression. In fact, renal cAMP levels were decreased by HWI alone, and salt loading did not have an effect on the action of HWI. It is also notable that high sodium intake did not change urine volume or urine osmolality, suggesting that abundant water drinking may counteract the effect of salt loading.

In a non-randomized human study, Higashihara et al. speculated that the increased urinary sodium in patients with HWI may be a result of high sodium intake, although SBP in HWI group patients was not different compared with a free water intake group [16]. Therefore, the major factor accelerating disease progression in HWI patients may not be increased blood pressure caused by high salt intake. In contrast, in our rat study, overload with high sodium caused higher SBP in addition to increased urinary sodium excretion, but the ameliorative effect of HWI was retained. Further, our correlation analyses showed that there was no correlation between KB% and sodium intake, KB% and U-Na, and SBP and KB%. The clinical study by Higashihara et al. was performed in autosomal dominant PKD patients, whereas our present experiments were conducted using PCK rats as a gene orthologous model of human autosomal recessive PKD. In human autosomal dominant PKD, AVP is thought to be a detrimental factor for disease progression [9, 23, 24] and consequently, tolvaptan, an AVP V_2_ receptor antagonist, has been approved for use in patients. Since either increased water intake so as not to increase plasma AVP levels [5, 15] or tolvaptan treatment to antagonize the V_2_ receptor [25] had an ameliorative effect against PKD in PCK rats, we consider that this rat strain is a useful model of AVP-accelerated disease progression. Indeed, V_2_ receptor antagonists have efficacy in several animal models of renal cystic disease, including PCK rats [26, 27]. Therefore, a translational study using a PCK rat model to mimic the AVP blockade reaction in PKD may be an appropriate strategy. As a next step, it is preferable to use different rodent models of renal cystic disease to determine whether HWI also ameliorates disease progression in these animals, and to elucidate the influence of salt loading on the effect of HWI.

In conclusion, at least in this model rat strain in the current disease stage, a detrimental impact of high sodium intake on PKD was not proven. To address the issue of whether HWI inhibits or promotes disease progression in patients, a long-term randomized study may be required.
